# Relationship between Infant Feeding and the Microbiome: Implications for Allergies and Food Intolerances

**DOI:** 10.3390/children11081030

**Published:** 2024-08-22

**Authors:** Lourdes Herrera-Quintana, Héctor Vázquez-Lorente, Daniel Hinojosa-Nogueira, Julio Plaza-Diaz

**Affiliations:** 1 Department of Physiology, Schools of Pharmacy and Medicine, University of Granada, 18071 Granada, Spain; lourdesherrera@ugr.es (L.H.-Q.); hectorvazquez@ugr.es (H.V.-L.); 2 Biomedical Research Center, Health Sciences Technology Park, University of Granada, 18016 Granada, Spain; 3 Unidad de Gestión Clínica de Endocrinología y Nutrición, Laboratorio del Instituto de Investigación Biomédica de Málaga (IBIMA), Hospital Universitario de Málaga (Virgen de la Victoria), 29590 Málaga, Spain; daniel.hinojosa@ibima.eu; 4 Department of Biochemistry and Molecular Biology II, School of Pharmacy, University of Granada, Campus de Cartuja s/n, 18071 Granada, Spain; 5 Instituto de Investigación Biosanitaria IBS, GRANADA, Complejo Hospitalario Universitario de Granada, 18014 Granada, Spain; 6 Children’s Hospital of Eastern Ontario Research Institute, Ottawa, ON K1H 8L1, Canada

**Keywords:** microbiome, infant feeding, food intolerance, allergies, formula feeding, breastfeeding

## Abstract

Childhood is a critical period for immune system development, which is greatly influenced by the gut microbiome. Likewise, a number of factors affect the gut microbiome composition and diversity, including breastfeeding, formula feeding, and solid foods introduction. In this regard, several studies have previously demonstrated that breastfeeding promotes a favorable microbiome. In contrast, formula feeding and the early incorporation of certain solid foods may adversely affect microbiome development. Additionally, there is increasing evidence that disruptions in the early microbiome can lead to allergic conditions and food intolerances. Thus, developing strategies to promote optimal infant nutrition requires an understanding of the relationship between infant nutrition and long-term health. The present review aims to examine the relationship between infant feeding practices and the microbiome, as well as its implications on allergies and food intolerances in infants. Moreover, this study synthesizes existing evidence on how different eating habits influence the microbiome. It highlights their implications for the prevention of allergies and food intolerances. In conclusion, introducing allergenic solid foods before six months, alongside breastfeeding, may significantly reduce allergies and food intolerances risks, being also associated with variations in gut microbiome and related complications.

## 1. Introduction

### 1.1. Overview of Infant Feeding Practices and Recommendations

Healthy eating practices during the first years of a child’s life are essential for adequate development and growth [1]. Therefore, dietary patterns should be shaped during infancy to ensure a lifetime of health and well-being. However, the World Health Organization (WHO) estimates that undernutrition accounts for 2.7 million (45%) of all child deaths annually [2]. Primarily, the first two years are particularly crucial, with ideal nutrition during this period lowering morbidity and mortality, decreasing the risk of chronic diseases, and enhancing their overall development [3].

Globally, infant nutrition guidelines face significant barriers. The economic environment, such as poverty, can limit the availability of health care, food, and other resources essential to mothers and infants’ health [4]. Geographic isolation makes critical resources unavailable. A mother’s need to work outside of the home, the availability of family support networks, access to community healthcare workers, and her autonomy also affect infant nutrition [5]. Moreover, cultural factors also influence breastfeeding and other food habits [6].

In general terms, breastfeeding should be initiated within one hour of birth, being the exclusive feeding source during the first 6 months of life. After this period, the introduction of complementary foods (solids) is recommended along with continuing breastfeeding until 2 years of age or beyond, as proposed by WHO and United Nations International Children’s Emergency Fund (UNICEF) [2,7].

#### 1.1.1. Breastfeeding

It is widely acknowledged that breastfeeding is the best food source for infants, guaranteeing a child’s well-being and health [8]. In addition to being clean, safe, and containing antibodies that help protect against many common childhood illnesses, it is also highly nutritious [9]. During the first 6 months of a baby’s life, breastmilk provides all the energy and nutrients they need. Furthermore, throughout the first 6 months to one year of life, it continues to meet 50% to 70% of their nutritional requirements [10]. However, contrary to official recommendations, the exclusive breastfeeding of infants under 6 months of age is estimated to be fewer than 50% [8]. Breastfeeding practice has been associated with several benefits for both mother and infant. For instance, as a result of breastfeeding, children are more likely to perform well on intellect tests, and less probable to be overweight or obese [11] and to develop diabetes later in life [2,8,9,10]. On the other hand, breastfeeding is linked to reduced risk of breast and ovarian cancer in women [2,8,9,10].

#### 1.1.2. Formula Feeding

The infant formula may serve as an alternative to human milk as the unique basis of nutrition for infants (i.e., children under the age of 12 months) [12]. These formulas are designed in such a manner as to preserve a balance between macro- and micronutrients. There are three main types of infant formulas: usual infant formula (0–6 months), continuation formula (6–12 months), and toddler formula (13–36 months). Both large and small populations of mothers who are unable to breastfeed their infants are served by the infant formula [12]. Generally, the majority of guidelines recommend that infants who are formula-fed should use commercial infant formula until they reach 12 months of age, and that infant formula is not necessary beyond that age [13]. Regarding cow milk, it is recommended delaying its use until 9–12 months in order to reduce iron deficiency in children [14].

#### 1.1.3. Introduction of Solid Foods

Complementary foods introduction to infants is a critical step in their development and long-term health status [15,16]. Additionally, there is evidence that the order in which complementary foods are introduced, their variety, and repeated exposure to these foods contribute to the development of food preferences later in life [17,18,19]. According to the European Food and Safety Agency (EFSA), no precise age for complemental feeding can be established, as it is strongly influenced by the infant’s characteristics and development [20]. According to WHO, infant formula is considered a complementary food, which is not directly comparable with the recommendations of the European Society for Paediatric Gastroenterology, Hepatology and Nutrition (ESPGHAN) and EFSA, both excluding infant formula as a complementary food [20,21].

Solid food introduction at the appropriate period may be a modifiable factor to improve a child’s growth and nutritional status [22,23]. Approximately after the first 6 months of life, infant formula or breast milk is no longer sufficient to meet the infant’s energy and nutrient requirements, and hence, solid foods should be initiated [24]. The introduction of solid food very early (at or before 4 months) appears to be related to a higher body mass index (BMI) at an older age [25,26,27,28,29]. According to the American Academy of Pediatrics (AAP), the Canadian Pediatric Society, and the WHO, solid food should be introduced at the age of 6 months, while according to ESPGHAN, it should be introduced between the ages of 4–6 months [30,31,32,33,34,35].

### 1.2. Importance of the Gut Microbiome and Its Early Development

Approximately 500–1000 species are thought to comprise the microbiota or microbiome, which includes a full collection of microbes that live in a particular biological niche, including bacteria, viruses, and fungi [36,37,38,39]. Microbes in the gut influence several aspects of human health, including immune and metabolic functions [40,41], or neurobehavioral characteristics [42]. As part of the microbiome of the gut, host nutrients, xenobiotics, and drugs are metabolized, the intestinal mucosal barrier is maintained, immune responses are modulated, and protection from pathogens is provided [43]. Moreover, a healthy gut microbiome plays an essential role in the fermentation of nondigestible substrates such as endogenous intestinal mucus and dietary fibers. In this process, short chain fatty acids (SCFAs) and gases are produced by selective microbes [44], with acetate, propionate, and butyrate being the most commonly produced SCFAs [45].

The composition of the gut microbiome is influenced by several factors. At the early stages, some of these factors include the method of delivery (i.e., vaginal or caesarean), the diet during infancy (e.g., formula or breast milk feeds), or the use of antibiotics [46,47]. This development and composition of the human gut microbiome has garnered significant attention owing to the crucial function of microbes in maintaining host health [48]. Early development of the intestinal microbiome determines adult microbiome composition and, therefore, health [49].

Infant microbiome composition can be traced to any of the maternal source communities on average across all infants [50]. There are multiple niches for infants in all maternal source communities. The microbiome of infants is shaped by shared and niche-specific host/environment factors [50]. In this regard, and according to longitudinal studies, the microbial structure of infants changes significantly with the cessation of feeding (breast or formula) and, therefore, with the introduction of solid foods [51]. As a result of the weaning process, the gut microbiome composition becomes stable as the taxonomic groups shift and the gut microbiome diversity increases from an infant to an adult. This process is modulated by diet [48,52]. Upon incorporating solid foods into the diet, the microbiome evolves from a simple environment, populated by *Bifidobacterium* (microorganisms that digest human milk oligosaccharides (HMOs)), to one that is populated by *Bacteroides*, which can metabolize starches present in a complex dietary pattern [53]. Thus, during the first years of life, the interaction between host and microbiome plays an important role, because considerable changes in structure and abundance happen at this important stage of the infant’s development [49].

Based on the above, the present review aims to examine the relationship between infant feeding practices and the microbiome, as well as its implications on allergies and food intolerances in infants.

## 2. Infant Feeding and the Microbiome Development

### 2.1. The Human Milk Microbiome: Composition and Impact on the Organism

The composition of the human milk microbiome may be determined by many factors, including lactation stage, maternal BMI, age, and diet [54], as well as by parity, geographical location, socioeconomic status, antibiotic or probiotic use during pregnancy, and type of delivery [54,55]. Among the nine genera that comprise the “core” bacteriome of the human milk microbiome are *Streptococcus*, *Staphylococcus*, *Pseudomonas*, *Serratia*, *Corynebacterium*, *Ralstonia*, *Sphingomonas*, *Bradyrhizobium*, and *Propionibacterium*. Approximately half of the microbial community in milk is represented by them, although the abundance of these bacteria varies from one sample to another [56,57].

In addition, it must be highlighted that breast milk also contains prebiotics, immunological substances, and other microbiome-shaped compounds to provide the infant with its own microbiome, possibly affecting colonization patterns elsewhere in the body [58]. As a result of the human milk microbiome supplying nutrients and dictating the production of metabolites, children’s gut and respiratory microbiomes are shaped [58,59,60].

Compared to the skin and respiratory microbiome, the fecal microbiome shows a continuing buildup of microbial diversity, while the skin and respiratory microbiome typically exhibit a unimodal pattern with a high initial diversity of microbes that rapidly declines over the first week, followed by a gradual increase in diversity [50]. Previous research suggests that the respiratory and skin microbiome, which are known as low-biomass places, may be seeded by other (low density) sources in addition to maternal seeding (e.g., other caregivers, hospitals, etc.) [61,62,63,64,65].

### 2.2. Benefits of Breastfeeding on Gut Health

Evidence highlights the importance of the “window of opportunity” during early childhood as being crucial to the gut microbiome long-term [66]. Therefore, the composition and metabolism of the neonate’s gut microbiome may play a critical role in the development of allergies [67]. In early life, breastfeeding affects the gut microbiome by exposing the neonate to the milk microbiome, both directly and indirectly, by incorporating factors in the maternal milk such as HMOs, secretory immunoglobulin (Ig)-A, and antimicrobials [68].

According to previous studies, the diversity of fecal bacteria increases with age, indicating that the microbial community becomes more complex with time [62,69]. Exclusively breastfed infants have shown to have a lower microbiome diversity than formula-fed babies, who present a higher diversity in their gut microbiome, being more similar to that of older children [70,71,72]. Therefore, breastfeeding results in a low level of bacterial diversity due to the predominance of infant-type *Bifidobacterium*, which is beneficial for children’s health. Infants who are exposed to *Bifidobacterium* in their early years may experience fewer infections due to the healthy maturation of their immune systems [73]. Likewise, *Bacteroides* has been seen to be another beneficial bacterium during the neonatal period since it plays a significant and specific role in the mucosal immune system development [74]. The presence of *Bacteroides* is also related to an earlier maturation rate and increased gut diversity [75].

Additionally, it is possible that other components of human milk may mediate the infant gut microbiome when interacting with the human milk microbiome. Secretory IgA, for example, may neutralize or inhibit the colonization of the human milk microbiome within the infant gut; this association remains unclear at present [76]. A study has shown that approximately 40% of human milk bacteria are IgA-coated [77], which may stimulate their colonization in the infant’s digestive system [76,78]. Human milk bacteria and other microorganisms, such as bacteriophages, remain largely unknown regarding their functional capabilities [79,80].

Although fewer studies have been conducted, human milk has been demonstrated to have a substantial impact on both the taxonomic composition (species abundance) and the functional capacity (functional related genes) of the resident microbiome, as well as their output (metabolites) [81]. Based on studies utilizing shotgun metagenomic sequencing, which sequences all genetic material within a microbial community, breastfeeding has been related to an enrichment in functional genes involved in fatty acid biosynthesis, vitamin B synthesis, and oxidative phosphorylation [52,62]. Infants’ microbiomes who do not receive human milk, on the other hand, exhibit a greater abundance of functional genes involved in methanogenesis and bile acid synthesis [62], as well as amino acid and nucleotide metabolism [52], all of which are typical of the more developed microbiome in adults.

### 2.3. Formula Feeding: Implications in Microbiome and Related Possibilities of Formulation

Interestingly, many advances have been made in the composition of formulas to mimic breast milk. In addition to identifying more constituents of human milk and describing their physiological functions, novel formulas continue to be developed, and established formulas are constantly being modified [82]. As a result, infant formula is increasingly supplemented with functional ingredients that are also found in breast milk to enhance its health-promoting capabilities [12,83,84], such as docosahexaenoic acid, lactoferrin, vitamins, minerals, and others. Despite this, infant formula cannot contain immunological components, enzymes, or hormones found in breast milk [82].

On the other hand, infant formulas can be designed to modulate the gut microbiome by incorporating probiotics, prebiotics, and symbiotics. The predominant probiotic strains incorporated into infant formulas are derived from the fecal microbiome of infants [85]. In this line, probiotics like *Bifidobacterium* and *Lactobacillus* [86], or oligosaccharides (prebiotics), may selectively stimulate the growth or metabolic activity of potentially beneficial indigenous bacteria such as bifidobacteria [87]. Furthermore, *Streptococcus* and *Propionibacterium* represent additional promising strains for use as probiotics [85]. It is critical to note that ESPGHAN posits that the administration of formulas fortified with probiotics and/or prebiotics currently used in infant formulas does not give rise to concerns regarding growth or possible adverse effects [88].

### 2.4. Timing and Types of First Foods and Their Impact on Microbiome Diversity

In the first 1 to 3 years of life, the gut microbiome of an infant undergoes significant changes from a relatively simple niche with low richness and diversity to one that resembles that of an adult [62]. Currently, we do not know how the introduction of complementary food (diversity of diet and food choices), after a diet consisting exclusively of milk, specifically affects the structure, diversity, and taxonomy of gut microbes [89,90]. Introducing solid foods may lead to the development of a microbiome that resembles that of an adult. This is a result of the changing ratios of protein, fat, fiber, and carbohydrate in the diet [91]. The observation of these gut bacteria in the adult population suggests that the introduction of solid foods facilitates the maturation of the gut microbiome of the child, leading to a configuration similar to that of adults [92].

During exclusive breast milk or formula feeding, the infant’s intestinal microbiome is predominantly composed of bifidobacteria and enterococci. Following the introduction of solid foods into the infant’s diet, there is a notable decline in the levels of these microorganism, while the relative abundance of fiber fermenters, including *Lachnospiraceae*, *Bacteroidaceae*, and *Ruminococcaceae*, increases [93]. In a recent study, infants’ gut microbiome was found to differ greatly between individuals at the time of introduction of solid food [94]. A strong cohort effect was also observed, which was expected, since the composition of gut microbiome is identified to vary geographically [95]. Based on the literature, inter-individual variability decreased with increasing age after the introductory period [62]. Within the first few weeks following birth to one year of age (14 months in the Netherlands), alpha diversity has been shown to increase over time [62,96]. Alpha diversity refers to the diversity of the microbiome in a single sample. In terms of alpha diversity, there are a number of indices that reflect different aspects of the heterogeneity of a community [97]. Thus, multifactorial components affect the gut microbiome at an early age, which may have a crucial role determining the microbiome’s composition and health in later life.

## 3. Microbiome and Allergies

### 3.1. Allergies: Underlying Mechanisms and Links with the Microbiome

Allergy pathogenesis involves a variety of factors, including genetics, epigenetics, environmental factors, microecology, and the immune system [98]. It is believed that a wide variety of cytokines, chemokines, allergens, and microbial products contribute to the complex cellular processes mediated by the adaptative and innate immune systems [99]. Particularly, a significant increase in Ig-E is associated with allergies, which are defined by a T-helper 2 (Th2) hypersensitivity response [100,101]. This immune response is characterized by the expression of cytokines, such as interleukin (IL)-13, IL-5, IL-4, and IL-9, leading to an inflammatory response [100,101]. In this line, regulatory T cells (Tregs) have been extensively studied and treated as potential therapeutic tools [102,103], since they display phenotypic and functional differences based on tissue location, differentiation status, disease state, and activation [104,105,106,107], enabling them to have diverse roles in allergy pathogenesis, among other processes [108,109].

For its part, the intestinal epithelium has a critical function in maintaining bodily homeostasis by serving as a protective barrier between the internal organs and the luminal microbiome [110,111]. Mucosal inflammation is characterized by the disruption of the intestinal epithelial barrier. In this regard, an increased exposure to luminal microbes triggers a perpetuating inflammatory response [112]. As a result of inflammation of the intestinal mucosa, multiple mechanisms may contribute to junctional disassembly and disruption of the epithelial barrier [113,114,115,116]. Among these mechanisms are a decreased expression of various tight junction (TJ) and adherens junction proteins, an impaired vesicular trafficking of junctional components, or an altered assembly and contractility of junctional actomyosin cytoskeletons [113,114,116,117].

A number of variables can affect intestinal permeability, including the modification of the gut microbiome, epithelial damage, dietary factors, and mucus layer dysfunction [118]. A dysbiosis, which may be caused by environmental factors, including drugs or diet, impairs the function of the epithelial barrier and produces proinflammatory cytokines. As a result of this process, TJ integrity is damaged and gut permeability is increased, resulting in leaky gut syndrome [119]. Thus, gut microbiome dysbiosis, in addition to affecting the intestinal epithelial barrier integrity, can result in antigen entry into the bloodstream and abnormal immune system stimulation [120]. Hence, intestinal and systemic diseases have been associated with impaired intestinal barrier function [121]. This may explain, in part, the relationship between respiratory allergies and gut microbiome dysbiosis [122].

Furthermore, other links between the gut microbiome and immune system have been reported in the literature. For instance, the gut-associated lymphoid tissue is a complex network of immune cells located at the deepest section of gut barrier [123]. Dendritic cells (DCs), intraepithelial DCs, intraepithelial lymphocytes, macrophages, TCD4+ lymphocytes, Tregs, plasma cells, and B lymphocytes are contained within the lamina propria [123]. Another example is the differentiation of T-helper 1 (Th1) cells, which is induced by contact with bacteria in the early stages of development. Hence, and taking all the above into consideration, it has been postulated that allergies could be prevented by regulating the intestinal microbiome [100,101,124].

### 3.2. Childhood Allergies and Microbiome

Over the past few decades, childhood allergies have become an increasingly prevalent issue [125], with various environmental factors, apart from genetic predisposition, potentially contributing to their development, such as cesarean birth, dietary habits, and antibiotic use. These factors also have an influence on the gut microbiome [126,127]. Moreover, it is known that dysbiosis, characterized by an imbalance between the function and composition of the microbiome, results in a breakdown of gut homeostasis and the development of diseases [128].

**Figure 1:**
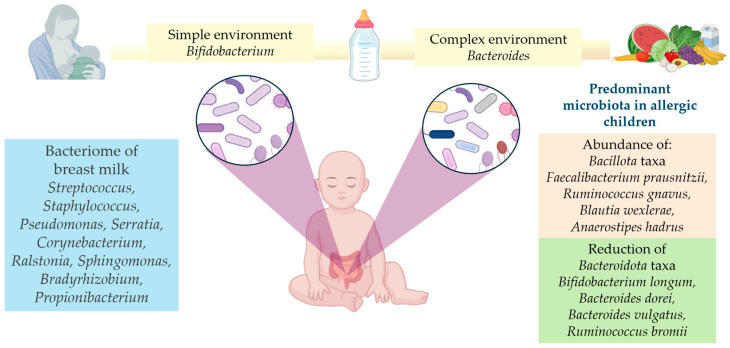
Changes in gut microbiome during infant feeding and in relation to allergies. Personal design.

According to previous reports, allergic children have a higher number of *Bacillota* taxa and a lower number of *Bacteroidota* taxa [129]. Furthermore, an increased abundance of *Faecalibacterium prausnitzii*, *Ruminococcus gnavus*, *Blautia wexlerae*, and *Anaerostipes hadrus* has been observed in children’s gut microbiome with respiratory allergies and food allergies, while *Bifidobacterium longum*, *Bacteroides dorei*, *Bacteroides vulgatus*, *Ruminococcus bromii*, and several other fiber-degrading species have been found to be significantly lower than in healthy controls [130,131,132,133,134,135]. Figure 1 represents the main changes in the gut microbiome during infant feeding and in relation to allergies.

On the other hand, evidence shows that microbe metabolites, particularly SCFAs formed after microbial fermentation of undigested fiber, are critical for maintaining epithelial integrity and stimulating immune tolerance [136,137]. Several studies have demonstrated that allergic subjects have low fecal levels of SCFAs [138,139,140], while children whose microbiomes lack genes related to fiber fermentation have a greater risk of developing allergic sensitization [130]. Moreover, a reduction in the ability to catabolize polysaccharides and the production of pro-inflammatory molecules may be contributing factors to allergic inflammation [127].

The relationship between microbiome and allergies may be mediated by a number of mechanisms; one of those mechanisms is immune system modulation, wherein the gut microbiome can produce anti-inflammatory cytokines, such as IL-10, and induce Tregs to modulate the immune system [141,142]. The Tregs are responsible for maintaining immune tolerance to harmless antigens, such as food proteins. As a result of a disturbed gut microbiome, individuals may experience decreased levels of Tregs and anti-inflammatory cytokines, resulting in a decrease in immune tolerance and the development of food allergies [143]. A further potential mechanism previously mentioned involves the gut microbiome, which contributes to maintaining the gut barrier function and prevents harmful substances from entering the bloodstream [144].

### 3.3. Microbiome Signature in Food Allergies

It is widely accepted that alterations in gut bacteria levels or diversity are responsible for the recent rise in food allergy rates [145,146]. During digestion and absorption, allergens from food are absorbed in the gut, primarily in the small intestine, which comprises a symbiotic microbiome [147]. As humans and the microbiome evolve together, symbiotic microbes will inescapably have a significant influence on human health. Because of the intestinal cavity’s high microbial diversity, immune cells react positively to allergens in this environment [148]. The human intestinal microbiome, particularly those in the ileum and colon, has been demonstrated to play a significant role in mucosal immunity in the intestinal tract through the promotion of local homeostasis and the regulation of the immune response to food allergens [149].

Recently, several landmark studies have revealed a microbiome signature in food allergies, particularly among children, which suggests that dysbiosis during childhood may predict the persistence of the disorder. Food allergy risk has been demonstrated to be influenced by the early colonization of the gut microbiome in babies [146]. It has been demonstrated that the intestinal microbiome composition of infants aged 3 to 6 months affects their milk allergy resolution at the age of 8 years. As compared with the children whose milk allergy persists, the children whose milk allergy resolves later have an intestinal bacterial composition that is enriched in *Clostridia* and *Bacillota* after birth [150].

As a result of consuming milk formula for 6 months, infants with cow’s milk allergies have a higher total bacteria count, particularly anaerobic bacteria [151]. In addition, there were higher levels of lactobacilli and lower levels of *Enterobacteriaceae* and *Bifidobacterium* in babies with cow’s milk allergies [151]. According to Bunyavanich et al. [150], *Clostridia* and *Bacillota* were particularly high in gut microbiome of infants whose cow’s milk allergy had solved by the age of eight [150]. Moreover, Fazlollahi et al. [152] found that the gut microbiomes of children suffering from egg allergy were more abundant with genera associated with *Lachnospiraceae* and *Ruminococcaceae* [152] than those of healthy controls.

Another immune reaction caused by food, specifically to eating gluten, is celiac disease. In wheat grains, gluten is the key storage protein, constituting an intricate combination of hundreds of associated but dissimilar proteins, mainly glutenin and gliadin [153]. Rye, barley, and oats contain similar storage proteins, also collectively referred to as “gluten”, but with some differences from wheat gluten (for instance, oats appear to be tolerated by a majority of celiac individuals) [153]. Gluten consumption triggers the symptoms of celiac disease, which is an inherited genetic disorder and can only be treated by maintaining a strict gluten-free diet [154]. However, a significant diminution in beneficial gut bacteria has been observed as a result of consuming a gluten-free diet, raising concerns about the potential risks [155]. Hence, there is particular interest in probiotic supplements because they can balance the gut microbiome and provide the required nutrients to remain healthy [156,157,158]. Furthermore, certain bacteria in the gut are known to break down gluten protein, thereby increasing or decreasing its toxicity [159]. A number of studies have demonstrated that the human gastrointestinal tract, including the proximal small intestine, contains gluten-degrading bacteria such as *Lactobacillus* spp. or *Rothia* spp. [160,161,162,163,164]. Thus, celiac disease patients may benefit from increasing or decreasing certain types of bacteria in their gut in order to reduce gluten toxicity [159].

### 3.4. Impact of Early Feeding Choices on Allergy Development

Approximately two to five percent of children have food allergies that can cause mild to severe allergic reactions. As such, the immune system develops sequentially as a result of a series of coordinated and timed events beginning during pregnancy and continuing throughout the first few weeks of postpartum [165]. Due to the modulation of the immune system by dietary components, there is also an effect of early diet on allergic disease susceptibility [166,167]. A number of specific dietary components, such as milk, peanuts, eggs, and fish, are usual allergens. Infants who consume those components may experience a tough response of immune system. Additionally, dietary compounds, including tryptophan, vitamins A and D, omega-3 polyunsaturated fatty acids, dietary fiber, and acetic acid, interact with various receptors to regulate immune homeostasis, including G protein-coupled receptors and nuclear receptors [166,167].

Recent studies have found that the delayed introduction of solids does not appear to protect against allergic diseases. Moreover, the late introduction of solid foods could increase allergic sensitization risk to foods, inhalant allergens, and celiac disease in children [168]. The development of tolerance might be influenced by the interaction of the mucosal immune system with the allergen at the appropriate age; the protective effects may be improved by breastfeeding during the transition to weaning [168].

Numerous randomized clinical trials have demonstrated that early introduction of allergenic complementary foods before the age of 6 months, along with continued breastfeeding, can greatly decrease food allergy risks [169,170,171,172]. According to a recent European evidence-based guideline on allergy prevention, the most effective time to introduce peanuts with complementary feeding is between the ages of 4–6 months [171]. Furthermore, the randomized clinical trial evidence indicates that complementary feeding introduction at the age of 4–6 months did not result in a reduction in the rate and duration of subsequent breastfeeding [170,173]. Nevertheless, this relationship between complementary feeding practices and allergic diseases in children has produced conflicting results, and it is not completely clear at present [174]. In general terms, and according to updated guidelines for allergy prevention, there is a consensus on the delayed introduction of major allergenic foods and a greater risk of food allergies [174]. In this line, a number of studies have shown that early introduction of allergenic foods, such as eggs and peanuts, can diminish the risk of food allergies in the predisposed child population. There is, however, no evidence to suggest that this may be applicable to the entire child population [170]. For one child to be protected from food allergies, 63 children should be introduced to allergenic foods at an early age [170].

## 4. Microbiome and Food Intolerances

### 4.1. Understanding Food Intolerances

A food intolerance occurs when the digestive tract becomes inflamed or when the food cannot be digested properly due to non-immune reactions involving toxic, pharmacologic, and unknown mechanisms (for example, lack of digestive enzymes). Unlike food hypersensitivity, which is a disorder of the immune system caused by specific proteins in food [175,176]. The diagnosis of food intolerances can take a considerable amount of time. Although food intolerance is not a life-threatening condition, it may cause the sufferer to feel extremely unwell and can have a significant impact on their work and social lives [177]. It has been estimated that up to 15–20% of the population suffers from food intolerance, depending on definitions and data collection methods [178]. In fact, the estimation of food intolerances two decades ago was also around to 20% of the population [179]. There are many people with food intolerance who report gastrointestinal symptoms, and 50–84% of patients with functional gastrointestinal disorders, such as irritable bowel syndrome, perceive their symptoms to be related to food intolerance [180,181,182].

As a result of their heterogeneous pathophysiological mechanisms, non-immunological adverse reactions to food can be classified broadly as host-independent and host-dependent [183]. The food intolerances reported by patients with irritable bowel syndrome are an example of undefined food intolerances that cannot easily be explained by current pathophysiological mechanisms [184]. Generally, food intolerance manifests in more than one organ or system; however, gastrointestinal symptoms, such as abdominal pain, bloating, abdominal distension, diarrhea, and flatulence, are quite common. It is important to note that non-allergenic food reactions are dose-dependent, as opposed to food allergies, in which traces of food allergens can trigger severe reactions [184].

### 4.2. Food Intolerances and Microbiome

Components from dietary food that are undigested by host enzymes convert to bacterial substrates and are transformed into metabolites such as SCFAs that regulate gut homeostasis [185]. Various factors contribute to the development of food sensitivities, including the dysfunction of the intestinal barrier, which can be induced by gut microorganisms and pathogens [186]. There are fermentable carbohydrates naturally occurring in a wide variety of foods, such as fermentable polyols and oligo-di-mono-saccharides and polyols. Patients with functional gastrointestinal symptoms, such as those suffering from irritable bowel syndrome, have been identified to be sensitive to this group of carbohydrates [187,188].

It is important to note that food intolerance is attributed to a variety of non-immune pathways, such as lactose maldigestion, which is caused by a primary or secondary β-galactosidase deficiency (lactase). Due to this, undigested lactose moves to the colon, where it is fermented by the microbes from the gut, causing the production of gas (methane, carbon dioxide, and hydrogen) and bloating [159,189]. Adult human gut *Bifidobacterium* abundance is determined by genetic variations associated with lactose intolerance and dairy consumption [190]. There is evidence that certain gut symptoms experienced by lactose intolerance patients might be the result of an abundance of *Bifidobacterium* in the gut rather than a direct reaction to lactose consumption [191]. The findings of the aforementioned study [191] support the initial reports suggesting that lactose-fermenting bacteria could be responsible for lactose intolerance symptoms [192,193,194]. Moreover, in eight studies, *Bifidobacterium* administration and lactose tolerance were examined; most (5/8) reported a positive effect of *Bifidobacterium* on managing lactose intolerance symptoms [195]. Research on the efficacy of acute and chronic *Bifidobacterium* consumption is limited. In order to determine the time required to improve lactose digestion and tolerance using *Bifidobacterium* on a long-term basis, more studies are required [195].

In recent years, extensive research has been made into gut microbiome disruptions characterized by intestinal symptoms. One of those receiving growing interest is small intestinal bacterial overgrowth (SIBO) [196]. SIBO’s prevalence is increasing worldwide, with the most common risk factor being diet and the Western diet being considered unfavorable for this condition [197]. Different bacteria overgrowth has been predominately recognized in SIBO, such as *Klebsiella pneumoniae*, *Escherichia coli*, *Prevotella*, *Streptococcus gramineus*, etc. [196]. Furthermore, a recent study showed that bacteria enriched in SIBO patients appears to be correlated with constipation, with the *Ruminococcaceae* group defined as core bacteria in SIBO [198].

## 5. Practical Implications

### 5.1. Recommendations for Infant Feeding

Recommendations for infant feeding and its impact on the infant microbiome should be focused on (I) providing exclusive breastfeeding for the first 6 months, as breast milk contains beneficial bacteria that help establish a healthy gut microbiome and provides prebiotics, which are food for the beneficial bacteria in the infant’s gut [199]; (II) frequent skin-to-skin contact between mother and baby, which can be used to transfer beneficial bacteria from the mother’s skin to the baby, promoting a healthy microbiome [200]; (III) feeding on demand, which helps to maintain an optimal balance of gut bacteria and supports the natural development of the infant’s immune system [201]; (IV) avoiding the use of antibiotics for the infant unless absolutely necessary and prescribed by a healthcare professional, as they can disrupt the gut microbiome [202]; (V) a varied and balanced maternal diet that can positively influence the composition of breast milk and, consequently, the infant’s gut microbiome [203]; (VI) introducing a variety of solid foods to further support the development of a diverse microbiome around six months of age, and including foods that are rich in fiber and natural prebiotics to nourish the beneficial bacteria [199]; (VII) avoiding formula unless necessary, as it does not provide the same microbiome benefits as breast milk—if possible, minimize formula use and continue breastfeeding [204]; (VIII) under the guidance of a healthcare professional, probiotics and prebiotics supplements might be recommended for the infant to support a healthy gut microbiome [205,206,207], mainly based on probiotics like *Lactobacillus rhamnosus*, *Bifidobacterium lactis*, and *Streptococcus thermophilus*, or common prebiotics as galacto-oligosaccharides, fructo-oligosaccharides, and inulin [208]; and (IX) donor milk usage, if the mother’s milk is not available. Pasteurized donor breast milk is preferable to formula, as it still contains beneficial components that support microbiome development [209].

### 5.2. Breastfeeding Encouragement and Support

Many mothers face challenges that make breastfeeding difficult. Support and encouragement can help overcome these barriers. Informing parents about breastfeeding benefits and teaching proper techniques can enhance breastfeeding success [210], whereas healthcare providers, including lactation consultants and nurses, play a crucial role in offering support and addressing concerns [211]. Moreover, peer support groups provide emotional encouragement and practical advice from other breastfeeding mothers [212].

Creating breastfeeding-friendly workplaces with facilities for pumping and storing breast milk can help mothers continue breastfeeding after returning to work [213]. In this line, policies that promote breastfeeding, such as the Baby-Friendly Hospital Initiative, can support breastfeeding initiation and continuation [214]. Other important encouragement techniques should be focused on (I) encouraging words and positive feedback, which can boost a mother’s confidence in her ability to breastfeed [215]; (II) helping mothers address specific breastfeeding issues, such as latching difficulties or low milk supply, which can improve outcomes [216]; and (III) regular check-ins with healthcare providers or support groups, which can provide ongoing support and guidance [217].

### 5.3. Choosing the Right Formula Based on Its Components

The most common ingredients used in the formulation are proteins, fats, carbohydrates, vitamins, and minerals [218]. As a source of protein, whey protein isolates, skim milk powder, whey protein concentrate, among others, made from milk, are commonly used. The most utilized fat source is vegetable fat, although animal milk fats can also be used [219,220]. Infant formulas are generally regulated to ensure they provide the essential nutrients infants need [12]. Although fundamental formulations are essential, there are considerable variations between countries and regions [221]. The specific composition will depend on the age range (0–36 months) and the presence of any underlying health conditions or special nutritional requirements [12].

The majority of infant formulas are produced from cow’s milk [85], although alternative animals’ milk, including goat and sheep, have also been extensively marketed as potential substitutes for allergy management and due to their better lipid profile [85,222]. Another option is the use of formulas derived from soy proteins, which have been demonstrated to be effective for infants with galactosemia or congenital lactase deficiencies. These formulas have been shown to treat colic and milk allergies [85], but their use is not recommended for infants under 6 months due to the presence of various components, including phytoestrogens [85]. Protein hydrolysate formulas are designed for infants intolerant or allergic to cow’s milk or soy-based formulas [85,223].

In the present day, manufacturers consider rice proteins to be a valuable raw material due to their high nutritional profile and hypoallergenic properties. A rice grain’s protein content varies according to genotype, climate, and cultural practices but is typically between 7 and 10% [224,225]. Hydrolyzed rice protein formulas have been developed, and they appear to be a viable alternative to cow’s milk protein formulas, both for families who wish to feed their children plant-based meals and for children with cow’s milk protein allergies [226]. The use of hydrolyzed rice protein formulas has become increasingly popular in some countries for the feeding of infants who have allergies to cow’s milk proteins [227]. Due to the legal framework of foods for special medical purposes, both cow’s milk protein allergy formulas and the quality of rice proteins allow infants and children to be fed without the risk of nutritional deficiencies [228].

Amino acid formulas represent an alternative for infants with a severe allergy to cow’s milk who exhibit a reaction to or refusal to consume adequate amounts of hydrolysate formula [85]. Finally, there are specific formulas designed for infants with a congenital metabolic defect, low birth weight, or those with an exceptional medical or dietary condition [229]. For example, additives are employed in anti-regurgitation formulas to reduce acid reflux [229]; the exclusion of amino acids, such as phenylalanine, from infant formulas for infants with phenylketonuria [229]; and an additional example is the formulation of formulas with a higher protein/energy ratio for infants with low birth weight or premature birth [230].

Formulas also include novel ingredients such as HMOs that inhibit microorganism adhesion to the intestinal mucosa. This inhibits pathogens growth, the expression of inflammation genes, and the production of bacteriocins and organic acids [231]. An HMO is a complex carbohydrate, often called a targeted prebiotic [232,233,234,235]. Research indicates that HMOs play a significant role in infant immunity. HMOs have a unique structure that provides targeted benefits, such as enhancing the development of the gut barrier and balancing the immune system [232,233,234,235]. Infant formula with HMOs is safe, well tolerated, and supports the child’s growth at the appropriate age [236,237]. Secondary outcomes indicated that consumption of HMO-supplemented formula were associated with lower parent-reported morbidity (particularly bronchitis) and medication usage (antipyretics and antibiotics) [236]. Furthermore, HMOs support a healthy gut microbiome, as well as promote brain growth and cognitive function [237,238,239,240].

In line with WHO, ESPGHAN promotes optimal nutrition and health for infants and young children, emphasizing breastfeeding as well as complementary feeding that is appropriate and timely [241]. ESPGHAN considered that continued breastfeeding during the second year of life may be a desirable goal for some families and children, depending on individual factors, personal preferences, and environmental factors [241]. It is recommended that complementary foods are introduced at a suitable age: Exclusive or full breastfeeding should be promoted for at least 4 months, and predominant/exclusive breastfeeding for 6 months is considered an appropriate goal. If a child or infant has an allergy to food, complementary foods with high allergenic potential (e.g., cooked egg, peanuts, or eggs) should be introduced in an age-appropriate manner when supplementation first begins at 4 months (17 weeks) [241].

### 5.4. Guidelines for Introducing Solid Foods

The introduction of solid foods represents a crucial element in the maintenance of optimal nutritional status, the prevention of potential allergic reactions, the reduction of the risk of disease, and the improvement of the modulation of the gut microbiome. Different aspects must be considered during the introduction of complementary foods, which typically occurs between 6 and 23 months of age, as this period is crucial for physical and cognitive development [242]:

(a) Choosing the adequate moment for every infant: In accordance with the recommendations of the AAP, WHO, and ESPGHAN, the introduction of solid foods is advised to commence between 4 and 6 months, based on the child’s developmental stage (e.g., the ability to sit in a chair and hold their heads up, interest in food, opening their mouths when approached with a spoon of food, putting their hands or toys in their mouths, loss of the tongue thrust reflex, etc.) [242,243]. It must be noted that an early introduction of solid foods may have negative effects. For instance, if the digestive and immune systems are not sufficiently developed, there are higher probabilities of choking, acute gastroenteritis, and upper respiratory tract infections [244].

(b) Food texture/consistency: Initial foods should be pureed, mashed, or semi-solid. At the age of 8–12 months, the introduction of foods that can be consumed with the hands is recommended. Ultimately, after 12 months of age, most children will have reached the same dietary texture options as the rest of the family [35,245]. During this period, the modification and exposure to a wide variety of textures is recommended to encourage the acceptance and enjoyment of food [93].

(c) Food quantity: The quantity, as well as consistency, should be increased gradually in accordance with the child’s growth and development [242]. However, the appropriate number of meals is dependent on the energy density of the food and the typical quantities consumed at each meal, which is typically two to three times a day until three to four times a day after 12 months [242].

(d) Food quality: Initial foods must be rich in iron and other minerals, such as zinc [35]. In this context, meat such as chicken or pork, eggs, or enriched cereals that can be mixed with breast milk, including oatmeal or rice, are recommended [242].

(e) Introduction of new foods and increasing acceptance: It is recommended that foods be introduced one at a time, with a recommended interval of 3 to 5 days between each introduction [35]. Due to the potential for allergic reactions, it is important to monitor for any adverse food-related responses. Extant evidence suggests that the early introduction of potentially allergenic foods, at approximately six months of age, may result in a gradual reduction in the risk of food allergy [93]. For enhancing tolerance to specific flavors, repeated exposures over a period of time between six and thirty six times are recommended [242]. The introduction of foods containing gluten, processed meats, fish, and shellfish should be carried out with caution due to the potential presence of components that may pose a significant health risk for the child [246,247].

(f) Some examples of appropriate first foods: soft pieces of banana, scrambled eggs, well-cooked pasta, well-cooked or mashed potatoes or peas, etc. They may help to prevent choking [248].

### 5.5. Future Research Directions

Food allergies and intolerances are more common in children than in other age groups. While food intolerances are more prevalent and less harmful, food allergies can be life-threatening. Therefore, a better understanding of the adverse effects of foods on functional disorders is needed [249]. Moreover, the dramatic global increase in the prevalence of these disorders requires effective strategies, considering the environmental factors, with emphasis on the study of the unknown underlying mechanisms, especially those related to the microbiome and immune system [126].

Although breastfeeding is the most recommended feeding method in infancy, some aspects, like the presence of allergens in breast milk or the effect of early introduction of allergenic foods, are still inconclusive. Thus, possible modifying factors (e.g., HMO and SCFA composition, the introduction of solid food, and immunologic factors) should be studied more in depth [250]. In this line, it has been hypothesized that regulating the composition of breastmilk, including the microbiome, could prevent allergic diseases early in life [251]. On the other hand, diet-derived metabolites determine the biologic effects of diet, and they vary according to different metabolic pathways in the host and in the gut microbiome, which may contribute to the heterogeneity observed when evaluating responses to diet interventions [252].

Microbiome research needs to be improved with appropriate experimental, methodological, and statistical designs. In this line, some recommendations have been made, including (I) selecting the adequate sampling frequencies, (II) using a combination of available methods in microbiome studies, (III) considering interactions between microorganisms and their hosts, or (IV) implementing a toolbox of technologies and more cultivation-based approaches into microbiome research [253]. Furthermore, different factors, such as the use of antibiotics or breastfeeding, must be considered in all trial designs, especially in those with longer duration and follow-up periods [254].

Although the functional understanding of the microbiome has grown considerably with the addition of metabolomics, metatranscriptomics and metaproteomics, and metagenomics, effort is still needed to develop new ex vivo assays targeting panels of entire microbiomes, simple microbial communities, or individual bacterium [255]. Furthermore, a computational approach integrating multiple data layers instead of evaluating individual data would allow the validation of mechanistic insights and new discoveries [256].

Based on the above, longitudinal studies can help elucidate the mechanisms through which the microbiome influences immune responses and the development of allergies. They can reveal how different feeding practices affect the microbiome’s composition and function over time. Moreover, results from these studies can inform guidelines on infant feeding practices, emphasizing the importance of breastfeeding for microbiome health. They can also lead to the development of probiotics or dietary supplements aimed at mimicking the beneficial effects of breastfeeding for formula-fed infants. Finally, findings from longitudinal research can shape public health policies and initiatives aimed at promoting breastfeeding and supporting mothers in breastfeeding practices [257,258].

## 6. Conclusions

Breastfeeding continues to be the most recommended method for infant feeding, with particular emphasis during the first six months of life, as it is associated with a gut microbiome rich in microorganisms that digest human milk. Introducing solid foods into an infant’s diet modifies this microbiome, evolving from a simple environment to one that is more complex. The early introduction (before the age of 6 months) of allergenic complementary foods, along with continued breastfeeding, could greatly reduce the risk of allergies and food intolerances. Individuals with food intolerances and allergies present different gut microbiome patterns, which could be both the cause and consequence of several related complications. Further research is needed to fully understand the role of the microbiome, the functional interactions, and its implication in food allergies and intolerance pathogenesis.

## Data Availability

Not applicable.
